# Positive Mental Health Literacy: A Concept Analysis

**DOI:** 10.3389/fpsyg.2022.877611

**Published:** 2022-04-14

**Authors:** Daniel Carvalho, Carlos Sequeira, Ana Querido, Catarina Tomás, Tânia Morgado, Olga Valentim, Lídia Moutinho, João Gomes, Carlos Laranjeira

**Affiliations:** ^1^School of Health Sciences of Polytechnic of Leiria, Leiria, Portugal; ^2^Hospital Center of Leiria – Hospital de Santo André, Leiria, Portugal; ^3^Centre for Innovative Care and Health Technology, Polytechnic of Leiria, Leiria, Portugal; ^4^Abel Salazar Institute of Biomedical Sciences, University of Porto, Porto, Portugal; ^5^Center for Health Technology and Services Research (CINTESIS), NursID, University of Porto, Porto, Portugal; ^6^Nursing School of Porto, Porto, Portugal; ^7^Hospital and University Center of Coimbra - Hospital Pediátrico, Coimbra, Portugal; ^8^The Health Sciences Research Unit: Nursing, Nursing School of Coimbra, Coimbra, Portugal; ^9^Escola Superior de Saúde Ribeiro Sanches, Lisboa, Portugal; ^10^Psychiatric Hospital Center of Lisbon – Hospital Júlio de Matos, Lisboa, Portugal; ^11^Research in Education and Community Intervention, Piaget Institute, Viseu, Portugal

**Keywords:** mental health literacy, mental processes, positive mental health, concept analysis, health literacy, personal autonomy

## Abstract

**Background:**

The positive component of Mental Health Literacy (PMeHL) refers to a person’s awareness of how to achieve and maintain good mental health. Although explored recently, the term still lacks a clear definition among healthcare practitioners.

**Aim:**

To identify the attributes and characteristics of PMeHL, as well as its theoretical and practical applications.

**Methods:**

Literature search (using the Medline and CINAHL databases) and review, covering the last 21 years, followed by concept analysis according to the steps described by Walker and Avant approach.

**Results:**

Positive component of Mental Health Literacy is considered one component of MHL, integrating positive mental health. The concept’s attributes include: (a) competence in problem-solving and self-actualization; (b) personal satisfaction; (c) autonomy; (d) relatedness and interpersonal relationship skills; (e) self-control; and (f) prosocial attitude. Four case scenarios (model, borderline, related and contrary cases) were used to clarify the antecedents (individual factors and social/contextual factors) and consequences (individual sphere; relational/social sphere; contextual/organizational sphere) of PMeHL.

**Conclusion:**

Positive component of Mental Health Literacy is considered a component of MHL, which deserves attention throughout the lifespan, in different contexts and intervention levels. Considering PMeHL as a multi-faceted and dynamic construct will help understand the mechanisms that improve mental health and promote healthy behaviors. Priority should be given to robust primary research focused on nursing interventions that enhance and sustain PMeHL in people and families.

## Introduction

Mental Health Literacy (MHL) is an evolving concept, originally conceptualized by Jorm et al. (1997) (Wei et al., 2015; Bjørnsen et al., 2017) and today recognized as a determinant of a population’s mental health (Jorm, 2012; Wei et al., 2013; Kutcher et al., 2016a; Bröder et al., 2017). MHL should not be limited to mental health professionals or people with mental health illnesses (Sweileh, 2021). Rather, MHL at the societal and community level is of great importance, as a means of promoting mental health and healthcare and achieving the economic, environmental, and social ambitions of the 2030 Agenda for Sustainable Development Goals (World Health Organization [WHO], 2019). Integrating MHL interventions into strategies of health promotion, disease prevention, and acute and chronic disease management will be essential to engage individuals in a person-centerd model that configures individual and holistic approaches (Pelletier and Stichler, 2014; Jorm, 2019).

While originally developed for adults, the concept of MHL has since been extended to adolescents (Morgado et al., 2021a), as the first onset of many mental disorders usually occurs in childhood or adolescence (Jorm, 2019). MHL has been recognized as an important factor in promoting the youth’s mental health, potentially benefiting both individual and public mental health (Kutcher et al., 2015; Wei et al., 2015; Marinucci et al., 2022). MHL can be further strengthened through educational initiatives (Kutcher et al., 2016a). A recent systematic review found a positive effect of school-based educational interventions on improving mental health literacy (MHL) in adolescents (Olyani et al., 2021). The evidence has also underlined the need to promote MHL throughout the life span and in different contexts (Morgado et al., 2021b), because it increases the quality of life of people (Jafari et al., 2021).

Mental health literacy consists of components related to the knowledge and abilities necessary to benefit mental health (Jorm, 2012; Kutcher et al., 2016b). Based on MHL research, Kutcher et al. (2016b, p.567) defined distinct but related components, the foremost being: “Understanding how to obtain and maintain good mental health.” This central element for health promotion—referred to as Positive Mental Health Literacy (PMeHL) (Bjørnsen et al., 2017)—is complemented by the following three components of MHL: the recognition of mental disorders; help-seeking efficacy; and help-seeking strategies (e.g., Jorm, 2012; Kutcher et al., 2013, 2015; Wei et al., 2013; Bjørnsen et al., 2018). Bjørnsen et al. (2017) emphasized that this conceptualization goes beyond previous notions of MHL as mere knowledge of mental disorders and proposed the concept of PMeHL.

Practice and research have mostly focused on mental ill-health; however, growing evidence demonstrates that supporting positive mental health has long-term benefits (PMH) (Bjørnsen et al., 2018; Teixeira et al., 2019). Note that PMeHL does not exist in a vacuum, but rather interacts with other factors such as personality traits, literacy skills, availability of information, and personal motivation.

Therefore, fully understanding and defining the concept of PMeHL is necessary. Although there has been increased research on the concept of MHL, few concept analyses, if any, have specifically addressed PMeHL (Bjørnsen et al., 2017). To fill this gap, we aim to describe the current definition of PMeHL based on the more recent literature incorporating well-grounded practices and potential measurement tools. We also propose an illustration of the concept analysis.

## Materials and Methods

This concept analysis was based on the framework proposed by Walker and Avant (2019). The method involves eight steps: (1) selecting the concept; (2) identifying the aim of the analysis; (3) identifying how the concept is used; (4) determining the concept’s defining attributes; (5) identifying model cases, where all attributes are exhibited; (6) identifying borderline, related and contrary cases, respectively, where most, part or no attributes are exhibited; (7) identifying determining antecedents and consequences; and (8) defining empirical referents.

### Database Search

A comprehensive literature search from 2000 to the present was conducted using two databases (Medline and CINAHL). The following search terms [MeSH] were used in combination: (Mental health literacy OR mental health*) AND (Positive mental health*) AND (Nursing OR Positive, Psychology) NOT (Child*). The studies published over the last 21 years reflect the changing perspectives and uses of the term.

The inclusion criteria for articles included in the analysis were: (1) a definition of PMeHL, a concept analysis, or a list of defining attributes; (2) instruments or methods to measure PMeHL; and (3) strategies to enhance PMeHL. We also included (4) position statements and definition papers addressing PMeHL. Only articles published in Portuguese and English were included.

### Data Source

**FIGURE 1:**
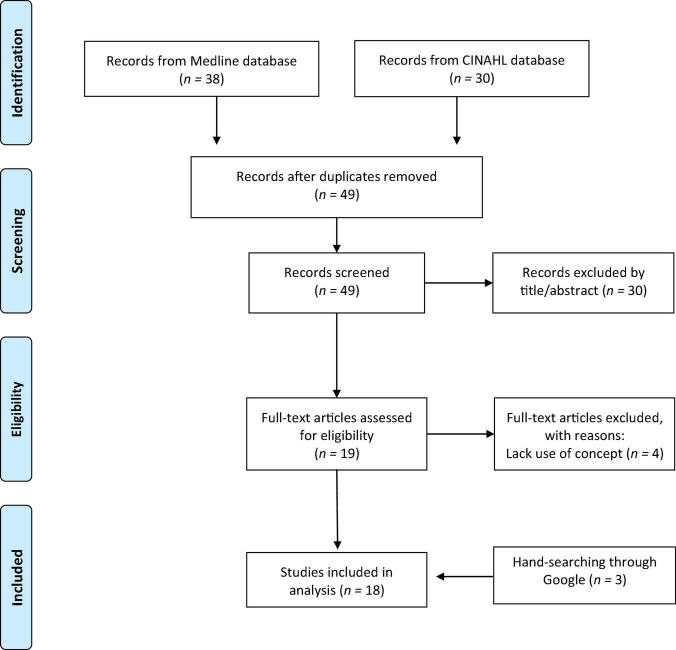
PRISMA flow diagram.

Figure 1 shows an overview of the article selection process. Of the 68 articles retrieved, 19 duplicates were excluded, and 30 titles/abstracts were deemed irrelevant, leaving 19 for full-text screening. Full-text screening excluded another four articles. Three additional articles were found with Google search engine, resulting in 18 included studies.

## Results

The 18 articles found during our search were critically analyzed to help identify PMeHL’s key attributes and develop case scenarios.

### Definitions and Uses of Concept

During the initial stage of concept analysis, Walker and Avant (2019) emphasize the need to identify the concept’s definitions and multiple uses.

Positive component of Mental Health Literacy is one component of MHL and refers to the knowledge and ability to obtain and sustain good mental health (Bjørnsen et al., 2017, 2018, 2019). This is in line with the previous broader concept of MHL, as defined by Kutcher et al. (2016b), which contains two aspects: knowledge about mental health, implying access to information and understanding that information; and the abilities to obtain and maintain good health, namely the skills needed to promote mental health and well-being and reduce the impact of mental illness in a Mental Health Continuum (Bjørnsen et al., 2018; Teixeira et al., 2019; Santini et al., 2020).

Positive component of Mental Health Literacy can include three dimensions of Positive Mental Health: (a) emotional (hedonic), covering the presence of positive affect and satisfaction with life; (b) social (eudaimonic), including both social functioning and connection to broader society; and (c) psychological (eudaimonic), covering intrapersonal and interpersonal functioning (Santini et al., 2020). Therefore, PMeHL can be understood as a process covering the search for knowledge and skills to obtain hedonic well-being (based on positive emotional states like happiness) and eudaimonic well-being (focuses on positive individual and social experiences and functioning) (Santini et al., 2020).

When making informed, effective decisions, PMeHL demands a strategy that appropriately conceptualizes the complex, dynamic nature of mental health literacy and includes the positive side of mental health for all participants.

### Defining Attributes

From the perspective of Walker and Avant (2019), defining attributes are critical characteristics that help differentiate related concepts and clarify their meaning. These characteristics are always included in a concept’s description. The key attributes of PMeHL are linked to fulfilling basic psychological needs (Deci and Vansteenkiste, 2004 cited in Bjørnsen et al., 2017), namely three inherent psychological demands for competence, relatedness, and autonomy. These are vital ingredients for proactivity, optimal development, and psychological health (Deci and Vansteenkiste, 2004 cited in Bjørnsen et al., 2017).

The multifactorial model of Positive Mental Health—based on a holistic perspective of health, wherein physical and mental health are intimately related—is also one of PMeHL’s critical characteristics (Lluch-Canut et al., 2013; Puig Llobet et al., 2020). Lluch’s conceptual model of PMH includes six factors, which are also used to define PMeHL attributes: personal satisfaction, prosocial attitude, self-control, autonomy, problem-solving, self-actualization, and interpersonal relation skills (Teixeira et al., 2022).

Furthermore, connection to mind and emotions encompasses the entire spectrum of basic cognitive, emotional, and psychological human experience, including the seven rights for mental health: a sense of belonging, control or mastery, self-esteem, meaning-making, values, motivation, and the need for secure relationships (Bjørnsen et al., 2018).

Therefore, the defining attributes of PMeHL are synthesized as follows:

(a) Competence in problem-solving and self-actualization—refers to a sense of mastery and efficacy in controlling one’s surroundings (e.g., handling stressful situations in a good manner, believing in oneself, having good sleep routines, mastering negative thoughts and experiencing school mastery) (Lluch-Canut et al., 2013; Bjørnsen et al., 2017; Santini et al., 2020); includes analytical capacity, decision-making ability, and flexibility to adapt to changes, demonstrating an attitude of continuous growth and personal development (Lluch-Canut et al., 2013; Bjørnsen et al., 2017; Santini et al., 2020).
(b) Personal Satisfaction—alludes to self-concept, self-esteem and self-acceptance, the ability to be satisfied with one’s personal life, and having an optimistic outlook on the future (Lluch-Canut et al., 2013; Santini et al., 2020). This attribute also includes interest and motivation that leads to emotional and psychological well-being (Bjørnsen et al., 2018; Santini et al., 2020) and feelings of happiness and life satisfaction (Santini et al., 2020).
(c) Autonomy—relates to personal security and self-confidence, as well as independence, a sense of personal choice or acting toward one’s own goals and according to one’s ideals, and self-regulation of one’s behavior (e.g., influencing daily living activities, acting out one’s wishes, making decisions based on one’s will, setting limits for one’s actions and setting limits for what is good for oneself) (Lluch-Canut et al., 2013; Bjørnsen et al., 2017).
(d) Relatedness and interpersonal relationship skills—includes the capacity to form interpersonal relationships, as well as empathy, which is defined as the ability to comprehend another’s feelings (Lluch-Canut et al., 2013). It covers the need to engage with, connect with, and care for others (e.g., having at least one good friend, being a good friend, feeling secure at home, feeling like a member of a community, and feeling worthwhile regardless of one’s own successes).
(e) Self-control—the capacity to manage stress/conflict, *via* emotional balance/control, and tolerate frustration, anxiety, and stress (Lluch-Canut et al., 2013).
(f) Prosocial attitude—an active predisposition toward society, including altruism and helping/supporting others, as well as acceptance of others and different social characteristics (Lluch-Canut et al., 2013). This predisposition is evidenced in terms of social functioning, such as contributing to the community, functioning well in the respective community and broader society, searching for social acceptance, actualization, and integration (Santini et al., 2020).

### Antecedents

Antecedents are the events or attributes that must exist prior to the occurrence of a concept (Walker and Avant, 2019). The 2000s brought enormous advances in “people’s knowledge, motivation, and competencies to access, understand, appraise, and apply information to make judgments and take decisions in everyday life concerning healthcare, disease prevention, and health promotion, and thus maintain and improve quality of life throughout the life course” (Sørensen et al., 2012, p. 3). From a salutogenic and socioecological perspective, the intervention of health care professionals takes place in different contexts and on a continuum, with special emphasis on health promotion and prevention.

The growing interest in searching for credible mental health information and being an active part in health decision-making has highlighted the person-centerd care model (Pelletier and Stichler, 2014). Many factors affect individual PMeHL, such as (a) individual factors (e.g., sex, race and ethnicity, educational level, mental health literacy, cognitive and emotional skills, feelings of vulnerability and resilience, physical and mental condition, self-determination and help-seeking behavior) and (b) social/contextual factors (e.g., cultural background, social and interpersonal skills, environmental events, exposure to health information and social resources in community) (Bjørnsen et al., 2017, 2018, 2019; Santini et al., 2020).

### Consequences

Consequences are occurrences or incidents that might happen because a concept is present and that frequently leads to new ideas or study areas for that concept (Walker and Avant, 2019). In this sense, consequences of PMeHL can be grouped into three spheres:

(a) Individual sphere

• Potential to promote, protect and restore mental health (Bjørnsen et al., 2017, 2018, 2019).
• Prevention of the development of mental disorders, with positive relevance to physical health (Iasiello et al., 2019; Mansfield et al., 2020; Puig Llobet et al., 2020).
• Maximization of hedonic and eudaimonic well-being (Santini et al., 2020).
• Improved satisfaction with life (Santini et al., 2020), connection, optimism and well-being (Jay et al., 2021).
• Increased autonomy in problem-solving ability and improved productivity, self-esteem, learning outcomes, resilience and motivation (Lluch-Canut et al., 2013; Sokołowska et al., 2018; Iasiello et al., 2019; Mansfield et al., 2020).
• Management of mental disorders and their treatments (Kutcher et al., 2016a; Sweileh, 2021).
• Increased help-seeking efficacy (Kutcher et al., 2016b).
• Improved health decisions to promote well-being (Bjørnsen et al., 2017).

(b) Relational/Social sphere

• Promotion of interaction, social well-being, and social functioning and satisfaction (Sokołowska et al., 2018; Iasiello et al., 2019; Mansfield et al., 2020) at the family, community or societal level (social contribution, social integration, social update, social acceptance, and social coherence) (Santini et al., 2020).
• Promotion of good mental health and personal wellness among nurses, leading to more human connections, positive feelings, work satisfaction, and better care provision (Jay et al., 2021).
• Promotion of the positive component of MHL among non-professional caregivers through mhealth intervention (Ferré-Grau et al., 2021).

(c) Contextual/organizational sphere

• Development of mental health promotion programs (Garmy et al., 2014; Bjørnsen et al., 2018; Teixeira et al., 2020) involving a mixture of professions [school social workers, teachers, psychologists, and other healthcare professionals] (Garmy et al., 2014; Diaz and Caboral-Stevens, 2021) and aligned with all development phases (Bjørnsen et al., 2018, 2019; Teixeira et al., 2019; Santini et al., 2020).
• Decreased stigma associated with mental illness (Kutcher et al., 2016a; Sweileh, 2021).
• Development of “organizational empathy” (Jay et al., 2021) as part of the nursing curricula. This competence facilitates the student’s transition to real workplace environments.

### Diagram Model of PMeHL

**FIGURE 2:**
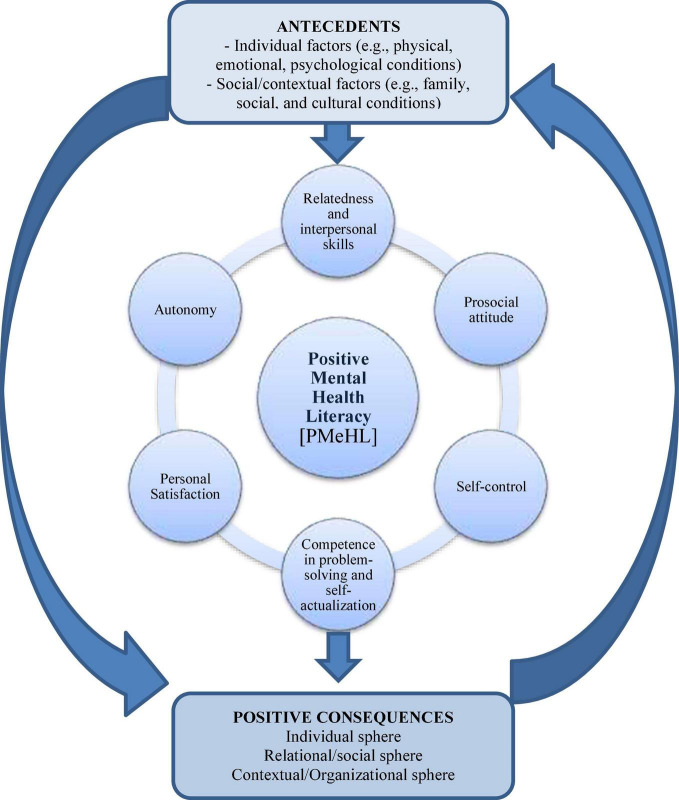
The link between PMeHL’s antecedents, attributes, and positive consequences is illustrated in this conceptual model.

Figure 2 illustrates the link between antecedents, attributes, and positive consequences in a comprehensive model of PMeHL.

### Case Scenarios

Cases help articulate the concept’s meaning. The model case displays all of the concept’s defining attributes, whereas the borderline case demonstrates most but not all of them, and the related case demonstrates only half of them. Lastly, the contrary case shows what the concept is not (Walker and Avant, 2019).

#### Model Case

According to Walker and Avant (2019), a model case is a circumstance in which all of the concept’s defining characteristics are present. In order to improve comprehension of PMeHL, we created a scenario to describe it with all of its identifying characteristics.

John is a 15-year-old son of divorced parents, has no siblings, and lives with his mother, with whom he has a good relationship. He feels safe at home. He has already flunked twice and is currently attending the 7th grade. He doesn’t identify much with his classmates because they are younger, although he demonstrates a willingness to participate in class activities and support his colleagues when they ask. He has a special friend with whom he shares his daily difficulties. To be with friends of the same age, who are already in high school, he spends some time at the skate park, which gives him personal satisfaction. His mother gets home quite late, for she has two jobs to balance the family finances. John reveals a good study-life balance and is able to set limits to his own actions. His remaining free time is spent on social media and the internet, which ends up being a source of information when he wants to learn more about Mental Health, problem-solving strategies and self-esteem promotion, and ways to deal with stress and anger. One of his favorite sites is https://mentalhealthliteracy.org/. He is accompanied by the Advanced Practice Psychiatric Nurse (PMH-APRN) from the school’s health service, whom he often turns to on his own initiative. Due to these regular encounters, he feels more relaxed, confident and valued after talking. In addition, these meetings help him deal with adversities and daily problems. Sometimes he analyses and discusses the information obtained from these sources with his colleagues and teachers. He considers himself a resilient person, but sometimes he feels pressured by the stereotypes and negative social visions around him. In this model case, John has adequate PMeHL to make his own decisions, as demonstrated by his accurate understanding of how to obtain and maintain good mental health.

#### Borderline Case

According to Walker and Avant (2019), a borderline case contains most of the defining attributes but not all of them. John is now 17 years old. After attending high school, he began to distance himself from friends, spending most of his time playing computer games, which gives him great satisfaction. For this reason, sleepless nights are increasingly common. His classmates begin to notice some anger and irritability in John’s behavior, revealing a lack of self-control. His school performance and results have deteriorated. In conversations with the PMH-APRN, he recognizes and expresses some depressive symptoms and loneliness. He accepts the idea of a specialized medical consultation, which his mother supports. John is ambivalent about taking sleep medication, as he fears becoming addicted. After sharing his concerns and receiving information, John decides to start his medication regimen. Whenever he has doubts or attributes an effect to the medication, he looks for information on the internet and asks the nurse for help. John receives support and understanding from his friends and feels comfortable sharing what is happening to him. This is an example of a borderline case, because John demonstrates all PMeHL attributes except one (e.g., self-control), but still demonstrates moderate PMeHL and makes some inappropriate health decisions.

#### Related Case

Related cases are instances in which the concept has some relation to the concept of focus, but does not contain every defining attribute (Walker and Avant, 2019). Upon entering higher education, John moves to another city, leaving his closest friends behind. As part of his new academic routines, he goes out at night and parties with new colleagues, which provides him personal satisfaction. These new routines interfere once again with his sleep pattern. He begins to have difficulty accomplishing the therapeutic regimen and sometimes voluntarily suppresses some doses. John becomes more anxious and testier with colleagues, which affects school performance and generates conflicts with classmates and housemates. He seeks help from the PMH-APRN, but does not feel the same empathy and understanding as in high school, and consultations become less frequent. John begins to question his symptoms and treatment regimen, and his medication adherence wavers. He searches for new ways to control symptoms and gets information from less reliable sources, without scientific support. Colleagues move away from him, due to some of John’s disruptive behaviors. This related case scenario lacks half the PMeHL attributes (e.g., relatedness, self-control, competence in solving problems).

#### Contrary Case

Mary is a 17 years-old college student who has been diagnosed with schizophrenia. She questions her diagnosis and the need for treatment, showing little insight into her situation. She is very reluctant to adhere to the proposed therapeutic regimen, which she abandons frequently, thinking herself cured. She does not seek help or information and is very unreceptive and sometimes reactive when health professionals offer her help. Her symptoms worsen and begin to interfere with her social relationships, family dynamics, and school performance, even leading her to drop out of school. This final case is an example of a contrary case because Mary lacks all of the defining attributes of PMeHL. She does not make the best judgments for her own care. She fails to control her mental illness, and because of her lack of PMeHL, she will suffer long-term effects.

### Empirical Referents

Empirical referents are measurable indicators of the concept’s occurrence (Walker and Avant, 2019). Across evaluated studies, only the 10-item Mental Health Promoting Knowledge (MHPK-10) scale measured knowledge of how to obtain and maintain good mental health (Bjørnsen et al., 2017). This valid and reliable one-dimension instrument was developed from the previous MHL definition, presented by Kutcher et al. (2016b), and the three dimensions of Basic Psychological Needs Theory (competence, autonomy, and relatedness) (Deci and Vansteenkiste, 2004 cit in Bjørnsen et al., 2017). To establish a solid grounding for the instrument, the items were based on a sound theoretical framework, adolescents’ opinions, and recognized experts (Bjørnsen et al., 2017). Respondents are asked to rank each item on a 6-point scale [do not know (0), completely wrong (1) … completely correct (5)], where a higher score implies greater knowledge. The MEST project (Bjørnsen et al., 2018, 2019) used the MHPK-10 to assess mental health-promoting education activities aimed at enhancing variables favoring mental health and better customizing these programs among Norwegian adolescents. Every item in the MHPK-10 is deemed appropriate and translatable to public health practice, since MHL is recognized as an outcome of mental health promotion actions (Bjørnsen et al., 2017).

## Discussion

In the current literature, PMeHL is a dynamic concept, viewed simultaneously as: a) an outcome of mental health promotion actions, with a positive connection to physical health, social interaction and functioning, problem-solving ability, productivity, self-esteem, learning outcomes, resilience and motivation (Lehtinen, 2008; Bjørnsen et al., 2017; Iasiello et al., 2019; Mansfield et al., 2020); and b) a resource or mediator of mental health and well-being, allowing the person to play a preventive role in the development of mental disorders (Bjørnsen et al., 2017).

In a broad sense, the antecedents that promote PMeHL represent the balance between the individual and the environment. These antecedents are clustered into two main categories: individual factors and experiences, and social/contextual interactions (including societal resources, and cultural values) (Bjørnsen et al., 2017, 2018, 2019; Santini et al., 2020). The defining attributes that characterize people with PMeHL include: (a) how they accept and value themselves; (b) how they control emotions by focusing on positive thoughts; (c) how they establish positive connections with others; (d) how they transform life’s disappointments into personal satisfaction; and (e) how they make their own decisions, revealing problem-solving skills. PMeHL is a phenomenon that manifests positive consequences, including the maximization of individual well-being on emotional, psychological and social domains (Santini et al., 2020). This potential to promote, protect and restore mental health protects the individual against several negative outcomes in both people with and without mental disorders (Bjørnsen et al., 2017, 2018, 2019). For Kutcher et al. (2016b), PMeHL increases the effectiveness in seeking help (knowing when, where and how to get good mental health care and developing the necessary skills for self-care) (Bjørnsen et al., 2018). Interestingly, age, gender, personality, educational, urban-rural, and cross-cultural differences in PMeHL may differentially affect rates of help-seeking across different settings/sectors, including health, education, and workplace context (Furnham and Swami, 2018).

According to Seedaket et al. (2020), two types of interventions are widely used to improve PMeHL: education-based and community-based interventions. The same authors concluded that stand-alone education-based interventions are likely more effective for improving MHL among adolescents. Nevertheless, community-based interventions could be more effective in later stages of the lifespan, namely adulthood and old age, because they include a wider range of outcomes, including mental health-related knowledge, quality of life, and social well-being (Castillo et al., 2019).

Because PMeHL is dynamic, it is important to assess a person’s level of comprehension, motivation for behavioral change, and changes in age or health condition. Individuals with greater PMeHL levels are more likely to engage in self-care and seek out resources from their family or social structure, community, or healthcare system. Individuals should be asked questions in an active voice that motivates them to ask more questions or seek more information while they are being educated (Parnell et al., 2019). Importantly, nurses and other healthcare professionals must realize that every encounter with individuals and family members is a chance to assess people’s mental health literacy and encourage learning and capacity to attain and maintain good mental health. Encouraging autonomy and personalizing treatment are two important aspects of effective nursing care.

Nurses must be educated on how to teach and incorporate mental health education into all elements of their normal care delivery, rather than consider it an additional duty (Parnell et al., 2019). Traditional methods of education, such as those based on written materials or delivered in a classroom setting, must be reconsidered. Other active learning strategies, such as roleplaying simulations, digital health interventions, and communication skills training (e.g., conveying compassion and empathy, offering hope and humor, assertiveness, and active listening techniques), are required in basic and continuing education (Parnell et al., 2019; Laranjeira and Querido, 2021).

Besides, a systematic approach to mental health promotion, such as MEST (Bjørnsen et al., 2018, 2019), suggests that school nurses, teachers and stakeholders collaborate and include positive MHL in mental health education. In addition, primary research focused on nursing interventions that enhance and sustain PMeHL in people and families should be prioritized. The findings of this concept analysis can be used to provide a conceptual framework for future study in this area.

### Limitations

There are a few limitations to this concept analysis. First, the literature search was thorough, but limited to two databases and the English and Portuguese literature; therefore, relevant publications may have been missed. Moreover, the established list of attributes, antecedents, and consequences may not be complete, and other existing PMeHL elements may have been excluded from the current concept analysis. Third, while the research focuses mostly on adolescents, PMeHL is a phenomenon that may occur in a variety of contexts (families, schools, streets, and workplaces) and health/illness type transitions, throughout the lifespan. Finally, the area is still dominated by a mental-illness perspective, with just a few measures used to examine PMeHL. Further studies should look toward creating new measures that are reliable, valid, and feasible, as well as testing measurement invariance on existing PMeHL measures across cultures. This might help understand cultural differences in mental health discourse and what it means to be positive mental health literate.

## Conclusion

The PMeHL concept is considered a component of MHL, integrating positive mental health, which deserves attention throughout the lifespan in different contexts and intervention levels. Emphasis on person-centerd care calls for a transformation of the mental-ill health paradigm (Whitaker et al., 2021). Thus, PMeHL can be operationalized in two ways: (a) as a resource or potential mediator between individual determinants and health(-related) outcomes and (b) as an outcome when attempting to decrease the impact of social/contextual inequalities on health(-related) outcomes. PMeHL is not restricted to a particular action and may alter as circumstances change. In sum, by approaching PMeHL as a multi-faceted and dynamic construct, we must learn more about the processes of improving mental health and promoting good behaviors.

## Author Contributions

All authors listed have made a substantial, direct, and intellectual contribution to the work and approved it for publication.

## Conflict of Interest

The authors declare that the research was conducted in the absence of any commercial or financial relationships that could be construed as a potential conflict of interest.

## Publisher’s Note

All claims expressed in this article are solely those of the authors and do not necessarily represent those of their affiliated organizations, or those of the publisher, the editors and the reviewers. Any product that may be evaluated in this article, or claim that may be made by its manufacturer, is not guaranteed or endorsed by the publisher.

## References

[B1] Bjørnsen H. N. Eilertsen M. E. Ringdal R. Espnes G. A. Moksnes U. K. (2017). Positive mental health literacy: development and validation of a measure among Norwegian adolescents. *BMC Public Health* 17:717. 10.1186/s12889-017-4733-6 28923031 PMC5604188

[B2] Bjørnsen H. N. Espnes G. A. Eilertsen M.-E. B. Ringdal R. Moksnes U. K. (2019). The Relationship between positive mental health literacy and mental well-being among adolescents: implications for school health services. *J. Sch. Nurs*. 35 107–116. 10.1177/1059840517732125 28950750 PMC7323733

[B3] Bjørnsen H. N. Ringdal R. Espnes G. A. Eilertsen M.-E. Moksnes U. K. (2018). Exploring MEST: a new universal teaching strategy for school health services to promote positive mental health literacy and mental wellbeing among Norwegian adolescents. *BMC Health Serv. Res.* 18:1001. 10.1186/s12913-018-3829-8 30594201 PMC6310949

[B4] Bröder J. Okan O. Bauer U. Bruland D. Schlupp S. Bollweg T. M. (2017). Health literacy in childhood and youth: a systematic review of definitions and models. *BMC Public Health* 17:361. 10.1186/s12889-017-4267-y 28441934 PMC5405535

[B5] Castillo E. G. Ijadi-Maghsoodi R. Shadravan S. Moore E. Mensah M. O. Docherty M. (2019). Community interventions to promote mental health and social equity. *Curr. Psychiatry Rep.* 21:35. 10.1007/s11920-019-1017-0 30927093 PMC6440941

[B6] Diaz D. Caboral-Stevens M. (2021). Health-promoting behavior and positive mental health of Filipino nurses in Michigan. J. Nurs. Pract. *Appl. Rev. Res.* 11, 20–26. 10.13178/jnparr.2021.11.01.1004

[B7] Ferré-Grau C. Raigal-Aran L. Lorca-Cabrera J. Lluch-Canut T. Ferré-Bergadà M. Lleixá-Fortuño M. (2021). A mobile app-based intervention program for nonprofessional caregivers to promote positive mental health: randomized controlled trial. *JMIR Mhealth Uhealth* 9:e21708. 10.2196/21708 33480852 PMC7864775

[B8] Furnham A. Swami V. (2018). Mental health literacy: a review of what it is and why it matters. *Int. Perspect. Psychol*. 7 240–257. 10.1037/ipp0000094

[B9] Garmy P. Berg A. Clausson E. K. (2014). Supporting positive mental health development in adolescents with a group cognitive intervention. *Br. J. Sch. Nurs.* 9 24–29. 10.12968/bjsn.2014.9.1.24

[B10] Iasiello M. van Agteren J. Keyes C. Cochrane E. M. (2019). Positive mental health as a predictor of recovery from mental illness. *J. Affect. Disord*. 251 227–230. 10.1016/j.jad.2019.03.065 30927584 PMC6487880

[B11] Jafari A. Nejatian M. Momeniyan V. Barsalani F. R. Tehrani H. (2021). Mental health literacy and quality of life in Iran: a cross-sectional study. *BMC Psychiatry* 21:499. 10.1186/s12888-021-03507-5 34641793 PMC8507341

[B12] Jay E.-K. Moxham L. Patterson C. (2021). An empathic shared learning community fosters positive mental health. *Aust. Nurs. Midwifery J.* 27:46. 10.3316/informit.885457445321713

[B13] Jorm A. F. (2012). Mental health literacy: empowering the community to take action for better mental health. *Am. Psychol.* 67 231–243. 10.1037/a0025957 22040221

[B14] Jorm A. F. (2019). “The concept of mental health literacy,” in *International Handbook of Health Literacy: Research, Practice and Policy Across the Lifespan*, eds Okan O. Bauer U. Levin-Zamir D. Pinheiro P. Sørensen K. (Bristol: Policy Press), 53–66.

[B15] Jorm A. F. Korten A. E. Jacomb P. A. Christensen H. Rodgers B. Pollitt P. (1997). “Mental health literacy”: a survey of the public’s ability to recognise mental disorders and their beliefs about the effectiveness of treatment. *Med. J. Aust.* 166 182–186. 10.5694/j.1326-5377.1997.tb140071.x 9066546

[B16] Kutcher S. Bagnell A. Wei Y. (2015). Mental health literacy in secondary schools: a Canadian approach. *Child Adolesc. Psychiatr Clin. N. Am*. 24 233–244. 10.1016/j.chc.2014.11.007 25773321

[B17] Kutcher S. Wei Y. Coniglio C. (2016a). Mental health literacy: past, present, and future. *Can. J. Psychiatry* 61 154–158. 10.1177/0706743715616609 27254090 PMC4813415

[B18] Kutcher S. Wei Y. Costa S. Gusmao R. Skokauskas N. Sourander A. (2016b). Enhancing mental health literacy in young people. *Eur. Child Adolesc. Psychiatry* 25 567–569. 10.1007/s00787-016-0867-9 27236662

[B19] Kutcher S. Wei Y. McLuckie A. Bullock L. (2013). Educator mental health literacy: a programme evaluation of the teacher training education on the mental health & high school curriculum guide. *Adv. Sch. Ment. Health Promot.* 6 83–93. 10.1080/1754730X.2013.784615

[B20] Laranjeira C. Querido A. (2021). Assertiveness training of novice psychiatric nurses: a necessary approach. *Issues Ment. Health Nurs.* 42 699–701. 10.1080/01612840.2020.1838008 33166221

[B21] Lehtinen V. (2008). *Building up Good Mental Health, Guidelines Based on Existing Knowledge. Publication of the Project: Monitoring Mental Health Environments (MMHE).* Jyväskylä: Gummerus Printing.

[B22] Lluch-Canut T. Puig-Llobet M. Sánchez-Ortega A. Roldán-Merino J. Ferré-Grau C. (2013). Assessing positive mental health in people with chronic physical health problems: correlations with socio-demographic variables and physical health status. *BMC Public Health* 13:928. 10.1186/1471-2458-13-928 24093443 PMC3853147

[B23] Mansfield R. Patalay P. Humphrey N. (2020). A systematic literature review of existing conceptualisation and measurement of mental health literacy in adolescent research: current challenges and inconsistencies. *BMC Public Health* 20:607. 10.1186/s12889-020-08734-1 32357881 PMC7195735

[B24] Marinucci A. Grové C. Allen K.-A. (2022). A Scoping review and analysis of mental health literacy interventions for children and youth. *Sch. Psychol. Rev.* 15. 10.1080/2372966X.2021.2018918

[B25] Morgado T. Loureiro L. Rebelo Botelho M. A. Marques M. I. Martínez-Riera J. R. Melo P. (2021a). Adolescents’ empowerment for mental health literacy in school: a pilot study on ProLiSMental psychoeducational intervention. *Int. J. Environ. Res*. 18:8022. 10.3390/ijerph18158022 34360315 PMC8345420

[B26] Morgado T. Costa T. Araújo O. Silva R. (2021b). “Interventions for better mental health literacy,” in *Handbook of Research on Assertiveness, Clarity, and Positivity in Health Literacy*, eds Almeida C. V. Ramos S. (Hershey, PA: IGI Global), 187–207. 10.4018/978-1-7998-8824-6.ch011

[B27] Olyani S. Gholian Aval M. Tehrani H. Mahdiadeh M. (2021). School-based mental health literacy educational interventions in adolescents: a systematic review. *J. Health Lit.* 6 69–77. 10.22038/jhl.2021.58551.1166 34258329

[B28] Parnell T. A. Stichler J. F. Barton A. J. Loan L. A. Boyle D. K. Allen P. E. (2019). A concept analysis of health literacy. *Nurs. Forum* 54 315–327. 10.1111/nuf.12331 30793314

[B29] Pelletier L. R. Stichler J. F. (2014). Patient-centered care and engagement: nurse leaders’ imperative for health reform. *J. Nurs. Adm.* 44 473–480. 10.1097/NNA.0000000000000102 25148401

[B30] Puig Llobet M. Sánchez Ortega M. Lluch-Canut M. Moreno-Arroyo M. Hidalgo Blanco M. À Roldán-Merino J. (2020). Positive mental health and self-care in patients with chronic physical health problems: implications for evidence-based practice. *Worldviews Evid. Based Nurs*. 17 293–300. 10.1111/wvn.12453 32762130

[B31] Santini Z. I. Torres-Sahli M. Hinrichsen C. Meilstrup C. Madsen K. R. Rayce S. B. (2020). Measuring positive mental health and flourishing in Denmark: validation of the mental health continuum-short form (MHC-SF) and cross-cultural comparison across three countries. *Health Qual. Life Outcomes* 18:297. 10.1186/s12955-020-01546-2 32887631 PMC7650216

[B32] Seedaket S. Turnbull N. Phajan T. Wanchai A. (2020). Improving mental health literacy in adolescents: systematic review of supporting intervention studies. *Trop. Med. Int. Health* 25 1055–1064. 10.1111/tmi.13449 32478983

[B33] Sokołowska E. Kluczyńska S. Zabłocka-Żytka L. Wojda-Kornacka J. (2018). The “PsychoŻak” program - an example of using positive concepts of mental health in practice. *Psychiatr. Pol.* 52 157–164. 10.12740/PP/76732 29704422

[B34] Sørensen K. Van den Broucke S. Fullam J. Doyle G. Pelikan J. Slonska Z. (2012). Health literacy and public health: a systematic review and integration of definitions and models. *BMC Public Health* 12:80. 10.1186/1471-2458-12-80 22276600 PMC3292515

[B35] Sweileh W. M. (2021). Global research activity on mental health literacy. *Middle East Curr. Psychiatry* 28:43. 10.1186/s43045-021-00125-5

[B36] Teixeira S. Coelho J. Sequeira C. Lluch I. Canut M. T. Ferré-Grau C. (2019). The effectiveness of positive mental health programs in adults: a systematic review. *Health Soc. Care Community* 27 1126–1134. 10.1111/hsc.12776 31144395

[B37] Teixeira S. Ferré-Grau C. Sequeira C. A. Pires R. Carvalho J. C. Ribeiro I. (2022). Positive mental health in university students and its relations with psychological vulnerability, mental health literacy and sociodemographic characteristics: a descriptive-correlational study. *Int. J. Environ. Res. Public Health* 19 3185. 10.20944/preprints202202.0167.v1 35328870 PMC8949352

[B38] Teixeira S. Sequeira C. Lluch T. (2020). *Programa de Promoção de Saúde Mental Positiva para adultos (Mentis Plus+): Manual de Apoio.* Porto: A Sociedade Portuguesa de Enfermagem de Saúde Mental.

[B39] Walker L. O. Avant K. C. (2019). *Strategies for Theory Construction in Nursing*, 6th Edn. Upper Saddle River, NJ: Pearson.

[B40] Wei Y. Hayden J. A. Kutcher S. Zygmunt A. McGrath P. (2013). The effectiveness of school mental health literacy programs to address knowledge, attitudes and help-seeking among youth. *Early Interv. Psychiatry* 7 109–121. 10.1111/eip.12010 23343220

[B41] Wei Y. McGrath P. J. Hayden J. Kutcher S. (2015). Mental health literacy measures evaluating knowledge, attitudes and help-seeking: a scoping review. *BMC Psychiatry* 15:291. 10.1186/s12888-015-0681-9 26576680 PMC4650294

[B42] Whitaker L. Smith F. L. Brasier C. Petrakis M. Brophy L. (2021). Engaging with transformative paradigms in mental health. *Int. J. Environ. Res. Public Health* 18:9504. 10.3390/ijerph18189504 34574437 PMC8472367

[B43] World Health Organization [WHO] (2019). *HealthInSDGs.* Available online at: https://www.who.int/healthpromotion/conferences/9gchp/policy-brief4-health-literacy.pdf (accessed December 10, 2021).

